# A reduction of viral mRNA, proteins and induction of altered morphogenesis reveals the anti-HTLV-1 activity of the labdane-diterpene myriadenolide *in vitro*

**DOI:** 10.1186/s12866-014-0331-2

**Published:** 2014-12-24

**Authors:** Camila Pacheco Silveira Martins, Orlando Abreu Gomes, Marina Lobato Martins, Luciana Debortoli de Carvalho, Jaqueline Gontijo de Souza, Flavio Guimaraes Da Fonseca, Rodrigo Gonçalves Silva dos Santos, Margareth Spangler Andrade, Carlos Leomar Zani, Elaine Maria de Souza-Fagundes, Edel Figueiredo Barbosa-Stancioli

**Affiliations:** Laboratório de Virologia Básica e Aplicada (LVBA), Departamento de Microbiologia, Instituto de Ciências Biológicas, Universidade Federal de Minas Gerais, Avenida Antônio Carlos, 6627 Belo Horizonte, Minas Gerais Brazil; Interdisciplinary HTLV Research Group – GIPH - Fundação HEMOMINAS, Belo Horizonte, Minas Gerais Brazil; Núcleo de Ciências Exatas – FACE – Universidade FUMEC, Belo Horizonte, Minas Gerais Brazil; Serviço de Pesquisa, Fundação HEMOMINAS, Belo Horizonte, Minas Gerais Brazil; Centro Tecnológico SENAI CETEC, Belo Horizonte, Minas Gerais Brazil; Centro de Pesquisas Renè Rachou, FIOCRUZ, Belo Horizonte, Minas Gerais Brazil; Departamento de Fisiologia e Biofísica, Instituto de Ciências Biológicas, Universidade Federal de Minas Gerais, Belo Horizonte, Minas Gerais Brazil

**Keywords:** HTLV-1, Diterpene myriadenolide, Antiviral activity, HAM/TSP, ATL

## Abstract

**Background:**

Human T-lymphotropic virus 1 (HTLV-1) has been associated with leukemia/lymphoma (ATL) and myelopathy/tropical spastic paraparesis (HAM/TSP), in addition to other inflammatory diseases as well as infection complications. Therapeutic approaches for HTLV-1-related pathologies are limited. The labdane diterpene myriadenolide (AMY) is a natural product that exhibit biological activities, such as anti-inflammatory and antiviral activity as reported for HIV and herpesvirus.

**Results:**

We demonstrated that this natural product was able to inhibit the expression of *gag-pol* mRNA and substantially reduced the expression of the structural proteins p19 and gp46. Comparison of treated and untreated cells shows that AMY alters both the morphology and the release of viral particles. The Atomic Force Microscopy assay showed that the AMY treatment reduced the number of particles on the cell surface by 47%.

**Conclusion:**

We demonstrated that the labdane diterpene myriadenolide reduced the expression of the structural proteins and the budding of viral particles, besides induces altered morphogenesis of HTLV-1, conferring on AMY a new antiviral activity that may be useful for the development of new compounds with specific anti-HTLV-1 activity.

## Background

Human T-lymphotropic virus 1 (HTLV-1) is the causal agent of adult T-cell leukemia (ATL), HTLV-1-associated myelopathy/tropical spastic paraparesis (HAM/TSP) and other inflammatory disorders that may develop after a variable period ranging from months to decades [1-5].

Decisions regarding ATL treatment should be based on the classification of ATL subtype, the prognostic factors at disease onset, comorbidities and the response to initial therapy [6]. There was a successful study combining zidovudine and interferon alpha for ATL and ATL-lymphoma [7], but the most recent novelty in drugs for ATL is a promising therapeutic molecule based on a zinc finger nuclease designed to recognize and disrupt the promoter function of the HTLV-1 LTR and specifically to kill HTLV-1-infected cells, which was tested in *in vitro* and *in vivo* models [8].

Since the discovery of HAM/TSP, various therapeutic approaches have been used for patients presenting unremitting myelopathic symptoms. However, treatment is mainly symptomatic, and therapeutic guidelines for HAM/TSP are missing mainly due to the lack of randomized double-blind controlled clinical trials [9]. Because induction of chronic inflammation in the spinal cord by HTLV-1-infected T-cells was recognized as the major pathogenic mechanism underlying HAM/TSP, anti-inflammatory and antiviral therapies have been tested [10], and some clinical benefit has been demonstrated for corticosteroids, mainly oral prednisolone and intravenous methylprednisolone [11], interferon-α [12] and IFN-β1 [13]. In a recent study, the evaluation of the *ex vivo* and *in vitro* effects of ascorbic acid and IFN-α treatment on PBMCs of seronegative, asymptomatic carriers and HAM/TSP patients demonstrated antiproliferative and cell death-inducing and immunomodulatory effects of high-dose ascorbic acid [9].

Plants are recognized for their ability to produce a wealth of secondary metabolites, and many species have been used for centuries to treat a variety of diseases [14]. Many of these natural products have been shown to have interesting biological and pharmacological activities and are used as chemotherapeutic agents or serve as the starting point in the development of modern medicines [15-18]. *Alomia myriadenia* Schultz-Bip. ex Baker (*Asteraceae*) is an herb occurring in the central regions of Brazil. The ethanol extract of *A. myriadenia* contains the labdane-type diterpene myriadenolide (12*S*, 16-dihydroxy-ent-labda-7, 13-dien-15, 16-olide). Labdane-type diterpenes are known to occur in terrestrial plants and marine organisms. They display interesting biological activities, such as antibacterial, antifungal, anti-inflammatory, antileishmanial, cardiotonic, cytotoxic and immunomodulatory activities, and show potential for use as new drugs [19-24]. Herein, we report evaluation of the antiviral potential of myriadenolide as a strategy for the development of new therapeutics to treat HTLV-1-infected patients. We show promising results for the regulation of viral messengers, expression of viral proteins, and viral morphogenesis, as evaluated with transmission electron microscopy (TEM) and atomic force microscopy (AFM).

## Results

### Measurement of AMY cytotoxicity

**Figure 1:**
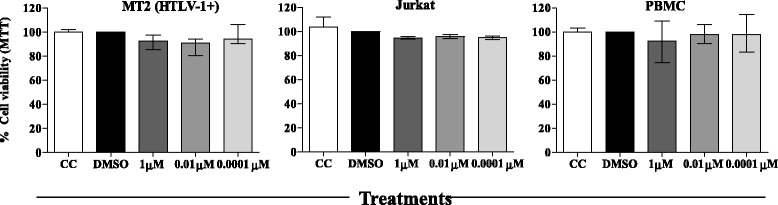
**Effect of myriadenolide on MT-2, Jurkat cells and PBMC viability.** Cells were treated with 1.0, 0.01 or 0.0001 μM myriadenolide for 24 hours. Cellular viability was inferred by MTT assay. All assays were performed in triplicate in at least two independent experiments. CC – Cell Control (Mock sample without DMSO and AMY). Average values with standard errors (error bars) are presented (GraphPad Prism 5).

To determine AMY cytotoxicity, MT2, Jurkat cells and human PBMC were treated with 1.0, 0.01 or 0.0001 μM AMY for 24 hours and the assay results are the mean of three independent experiments. For human PBMCs, the assay was performed in triplicate and with peripheral blood obtained from eight individuals. As demonstrated in Figure 1, treatment with different concentrations of AMY did not induce a significant reduction of cell viability in any of the cell types evaluated, compared to the diluent control (DMSO, 0.005%), showing the safety of this compound for the cell types tested, including human PBMCs, used as a control for normal cells.

### Quantification of viral mRNA in MT-2 cells after treatment with AMY

**Figure 2:**
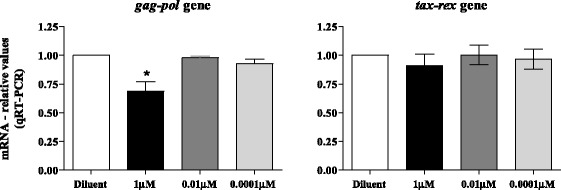
**Effect of myriadenolide on accumulation of mRNA** ***gag-pol*** **and** ***tax-rex*** **in MT-2.** Data represents the relative values of mRNA estimated by the method of relative quantification using standard curve established with cDNA from untreated MT2 cells (diluent control). Cells were treated with 1.0, 0.01 or 0.0001 μM myriadenolide for 24 hours. The assays were performed in two independent experiments. *Represents statistical difference (p = 0.026) between AMY 1 μM and diluent (GraphPad Prism 5; kruskall wallys with Dunn´s post-test).

The mRNA levels of the viral genes *gag-pol* (which encodes structural proteins and enzymes) and *tax-rex* (which encodes non-structural regulatory proteins) were quantified in MT-2 after 24 hours of AMY treatment using Real-Time PCR and were normalized to a cellular housekeeping gene (GAPDH). The analysis demonstrated that myriadenolide was able to inhibit the expression of *gag-pol* mRNA at 1 μM of AMY (Figure 2). However, inhibition of *tax-rex* mRNA expression was not observed at any concentration tested.

### Anti–HTLV-1 Activity of AMY in MT-2 cell line

**Figure 3:**
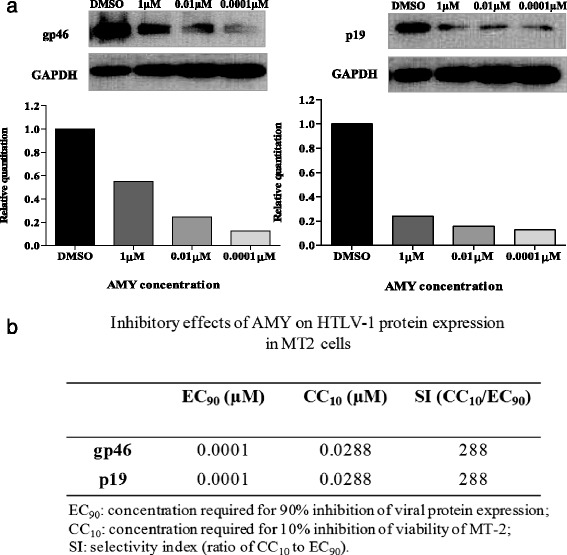
**Expression of viral proteins is reduced in MT-2 cells treated with myriadenolide.** MT-2 cells were treated with different concentrations of myriadenolide (1.0, 0.01 or 0.0001 μM) and viral protein expression was assessed. **(a)** Monoclonal primary antibodies were used to quantify protein expression: anti- gp46, p19 and GAPDH. Densitometric analysis was performed on ImageJ software to examine the level of HTLV-1 structural proteins normalized by GAPDH. **(b)** Selectivity index was calculated using CC_10_/EC_90_ as described in materials and methods. EC = effective concentration; CC = cytotoxic concentration.

Western blot assays were performed to determine the effect of myriadenolide on the expression level of structural protein p19 (matrix GAG protein) and gp46 (surface ENV protein) in MT-2 cells after 24 hours of AMY treatment at varying concentrations (1.0, 0.01 and 0.0001 μM). MT-2 cells treated with AMY showed a reduction in the viral proteins tested (Figure 3a). It was interesting to observe that for matrix protein p19 and ENV protein gp46, all AMY concentrations clearly decrease the level of protein expression, even at the lowest drug concentration.

In our assays, to determine the selectivity index of AMY, we choose to use the ratio CC_10_/EC_90_ (cytotoxic concentration – cytotoxicity activity that reduced the cell viability in 10%/effective concentration - concentration that reduce the viral protein expression in 90%), as this is pharmacologically more accurate in evaluating how safe a compound is as a drug [25]. The EC_90_ value of AMY to reduce levels of gp46 and p19 was 0.0001 μM, whereas its 10% cytotoxic concentration (CC_10_) was 0.0228 μM. Consequently, its selectivity index based on the ratio CC_10_/EC_90_ was 288 (Figure 3b). These results suggest that AMY has antiviral activity interfering with the expression of structural proteins of HTLV-1.

### Evaluation of AMY antiviral activity in MT-2 cells using transmission electron microscopy (TEM) and atomic force microscopy (AFM)

**Figure 4:**
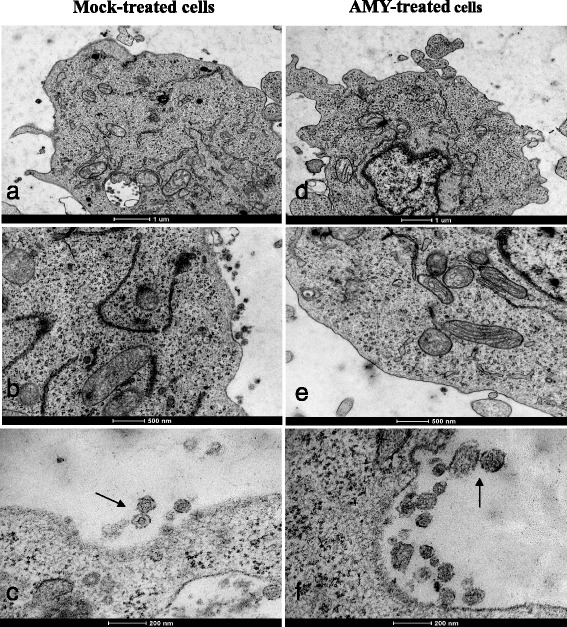
**Transmission electron microscopy of MT-2 cells treated with AMY.** MT-2 cell suspensions were cultivated with or without 1 μM of AMY. Twenty-four hours post-incubation, cells were stained with uranyl acetate and examined under a Tecnai G^2^ F20 electron microscope (FEI, USA). Panels **a** to **c** represent MT-2 untreated cells and D to F are MT-2 cells after AMY treatment. Viral budding is reduced in AMY treated cells when compared to mock-treated cells **(panels a, b, d and e)**. HTLV-1 typical particles are seen when cells are untreated (panel **c**; arrow) whereas HTLV-1 atypical particles (panel **f**; arrows) are seen in AMY treated cells. Scale bars are represented in each panel.

**Figure 5:**
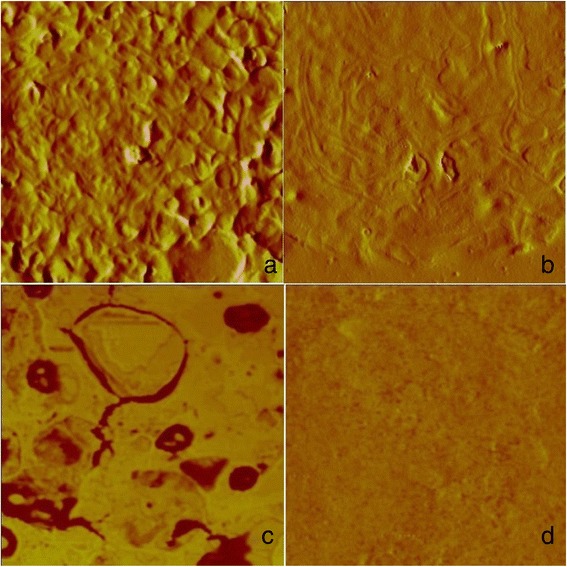
**AFM cell surface scanning of MT2 cells treated with 1 μM and 0.001 μM AMY.** The AFM images are phase and amplitude images and the regions scanned included both cells and matrix, and emphasis was placed on the cell surface. **(a)** 8.5 μm amplitude image showing the untreated MT-2 cell; **(b)** 8.5 μm image of MT-2 cell treated with 0.001 μM AMY; **(c)** 3.6 μm phase image of untreated MT-2 cells showing budding events with many virus particles embedded into a matrix; **(d)** 3.6 μm phase image of MT-2 cells treated with 1 μM AMY showing a flat cell surface with no virus budding.

The TEM and AFM analyses of MT-2 cells were performed 24 h post-incubation in the presence or absence of 1 μM AMY (Figure 4). Comparison of treated and untreated cells by TEM shows that treatment with AMY alters both the release of viral particles and the morphology (emphasized in Figure 4(b)/4(e) and 4(c)/4(f), respectively). However, we do acknowledge that the TEM may not be able to precisely determine whether these are actual complete virus particles or just empty capsids. Nonetheless, this observation was corroborated using AFM (Figure 5), and because this technique favors counting the number of particles per field evaluated, it was shown that AMY treatment reduced the number of particles on the cell surface by 47%. Of the 412 virus particles quantified by TEM, 229 viral particles were measured in cells treated with AMY and 183 in untreated cells. In the untreated cells, the virus particles ranged from 53 to 212 nm (mean 90.6 nm), and in the treated cells, they ranged from 41 to 171 nm (mean 91.55 nm). Using AFM, a total of 158 virus particles were measured (55 and 103 particles in AMY treated and untreated cells, respectively) and the size of viruses ranged from 75.8 to 273.6 nm (mean 148.9 nm) in untreated cells, whereas the sizes ranged from 42.4 to 175.5 nm (mean 97.9 nm) in AMY treated cells.

## Discussion

HTLV-1 is responsible for inducing a severe myelopathy and leukemia in humans, in addition to a number of other inflammatory pathologies for which treatment and therapeutic protocols are still ineffective. Consequently, HTLV-1 is responsible for a significant burden of morbidity and mortality [26]. In the present study, we address the possible antiviral activity of the labdane-type diterpene myriadenolide (12*S*, 16-dihydroxy-ent-labda-7, 13-dien-15, 16-olide) for HTLV-1. The antiviral activity of the terpenes has been reported for HIV [27,28] and herpesvirus [29], among other viruses.

HTLV-1 mainly infects CD4 T cells and deregulates their differentiation, function and homeostasis, which may contribute to the pathogenesis of HTLV-1 inducing chronic inflammatory diseases [30]. HTLV gene products are engaged in dynamic activating and antagonistic interactions with host cells, mainly driven by the Tax [31] and HBZ proteins [32]. However, recent mapping of the HTLV-1 and HTLV-2 host-pathogen interactome showed that other viral proteins, including Gag and Env from both viruses, are implicated in a diverse set of cellular processes, such as the ubiquitin-proteasome system, apoptosis, multiple cancer pathways and the Notch signaling pathway [33]. Naturally infected lymphocytes produce virtually no cell-free virions *in vivo*, and HTLV-1 is transmitted cell-to-cell through the formation of virological synapses, which are formed between an infected source cell and a susceptible target cell and play an important role in the spread of virus in the host [34]. It has also been demonstrated that there is a rapid transfer of enveloped HTLV-1 particles across virological synapses [35], reinforcing the idea that the glycoprotein Env is required for HTLV-1 infectivity [36]. Before these events, important early steps in the HTLV-1 assembly pathway include genome recognition (Gag-RNA interactions), as well as Gag-Gag and Gag-cellular protein interactions. The Gag polyprotein is composed of three domains: matrix (MA - p19), capsid (CA - p24) and nucleocapsid (NC - p15). In later steps upon budding or immediately after immature particle release, proteolytic cleavage of Gag polyproteins takes place and results in virus particle core maturation, showing that Gag coordinates assembly and viral budding [37-39]. Taken together, this information about morphogenesis, viral spread and virus-cell regulation introduces new concepts to target drugs for HTLV-1.

In this context, our results first revealed that the myriadenolide compound was able to reduce the accumulation of *gag-pol* mRNA at 1 μM after 24 hours of treatment (although no variation was seen for *tax-rex* mRNA in any concentration tested). HTLV-1 requires regulated gene expression from unspliced and alternatively spliced transcripts for efficient replication and persistence, being able to export intron-containing mRNA’s to cytoplasm for subsequent translation, function related to the viral protein Rex [40]. Rex phosphoprotein acts posttranscriptionally by preferentially binding, stabilizing, and selectively exporting the unspliced and incompletely spliced viral mRNA from the nucleus to the cytoplasm, essentially regulating production of the virion components [41]. Experiments conducted with transient transfection of 293T cells with the HTLV-1 plasmid, as well as newly HTLV-1 infected human PBMCs, clarified that incompletely spliced (*env*) and doubly spliced transcripts (tax/rex) are not generated at the same rate from full-length transcripts (*gag-pol*) [42]*.* Other important point is that unlike the *gag/pol* and *env* transcripts encoding the structural and enzymatic proteins, the efficient expression and cytoplasmic export of the alternatively spliced regulatory and accessory transcripts are not directly dependent on Rex [40]. However, the exact mechanism supporting the distinct activity observed upon AMY treatment for *gag-pol* and *tax-rex* transcripts remains to be determined. Nonetheless, we could speculate that phenomenon is based on differential gene expression regulation and Rex dependence for stability and transport of the both transcripts.

We observed a significant reduction on p19 and gp46 protein expression when using three different AMY concentrations (1.0, 0.01 and 0.0001 μM). Curiously the decreasing concentrations of AMY have increasing inhibitory activity on HTLV-1 antigen expression in MT2 cells (Figure 3a). This represents the hormetic dose-response effect (hormesis), in which a low dose can define the therapeutic zone (the intended effect, in our case, antiviral properties). Hormesis is a dose response relationship in which effects at low doses are opposite to those at high doses. Recent relevant reports analyzing hormetic dose responses indicates that this phenomenon can have specific mechanisms mediated by different receptors and signaling pathways, having biological diverse effects in distinct doses [43-45]. Thus, hormesis could explain why the lowest concentration of AMY (0.0001 μM) was best at reducing viral protein expression. Nonetheless, we did not see the same effect when analyzing transcript levels for the same proteins. At this point we still do not have a verifiable explanation to this otherwise biological effect.

The results also emphasize that AMY demonstrated no cytotoxicity when evaluated at concentrations up to 1 μM, as previously described by our group [24]. The selectivity index (288) showed that reduction of viral protein expression occurs with low toxicity. In drug discovery, it is desirable to have a high therapeutic/selectivity index (i.e., maximum antiviral activity with minimal cell toxicity). An index higher than 50 is considered as indicative of highly active compounds [46].

In concert with TEM, which clearly showed differences in particle morphology in treated and untreated cells (Figure 4), we used scanning AFM in air tapping mode to obtain more reliable results for viral particle measurement and number of budding particles. For untreated cells, the viruses ranged from 75.8 to 273.6 nm (mean 148.9 nm), and in treated cells, they ranged from 42.4 to 175.5 nm (mean 97.9 nm). Experiments using TEM and AFM proved that treatment of MT-2 for 24 hours using 1.0 μM myriadenolide was able to reduce the amount of virus at the cell surface (approximately 47%) and to modify the morphogenesis of viral particles, in accordance with the protein inhibition observed in Western blot assays.

## Conclusions

These results showed promising anti-HTLV-1 activity, mainly inhibition of Gag and Env protein expression and interference in viral budding and morphogenesis. It may be hypothesized that disruption in viral replication functions could reduce viral spread in infected hosts, suggesting a potential for AMY to be used as an anti-HTLV-1 therapeutic drug.

## Methods

### Cells

In this study, MT-2, an HTLV-1-infected T-cell line, and uninfected Jurkat T-cells were used. For the viability assays, human peripheral blood mononuclear cells (PBMCs) from seronegative blood donors were also used. Written informed consent was obtained, and this study was approved by the Ethics Committee of UFMG and Fundação HEMOMINAS. The cells were maintained in RPMI-1640 medium supplemented with 10-20% v/v fetal bovine serum (Life Technologies), 2 mM L-glutamine, 100 U penicillin mL^−1^ and 50 μg gentamicin mL^−1^ (SIGMA-ALDRICH).

### Myriadenolide

**Figure 6:**
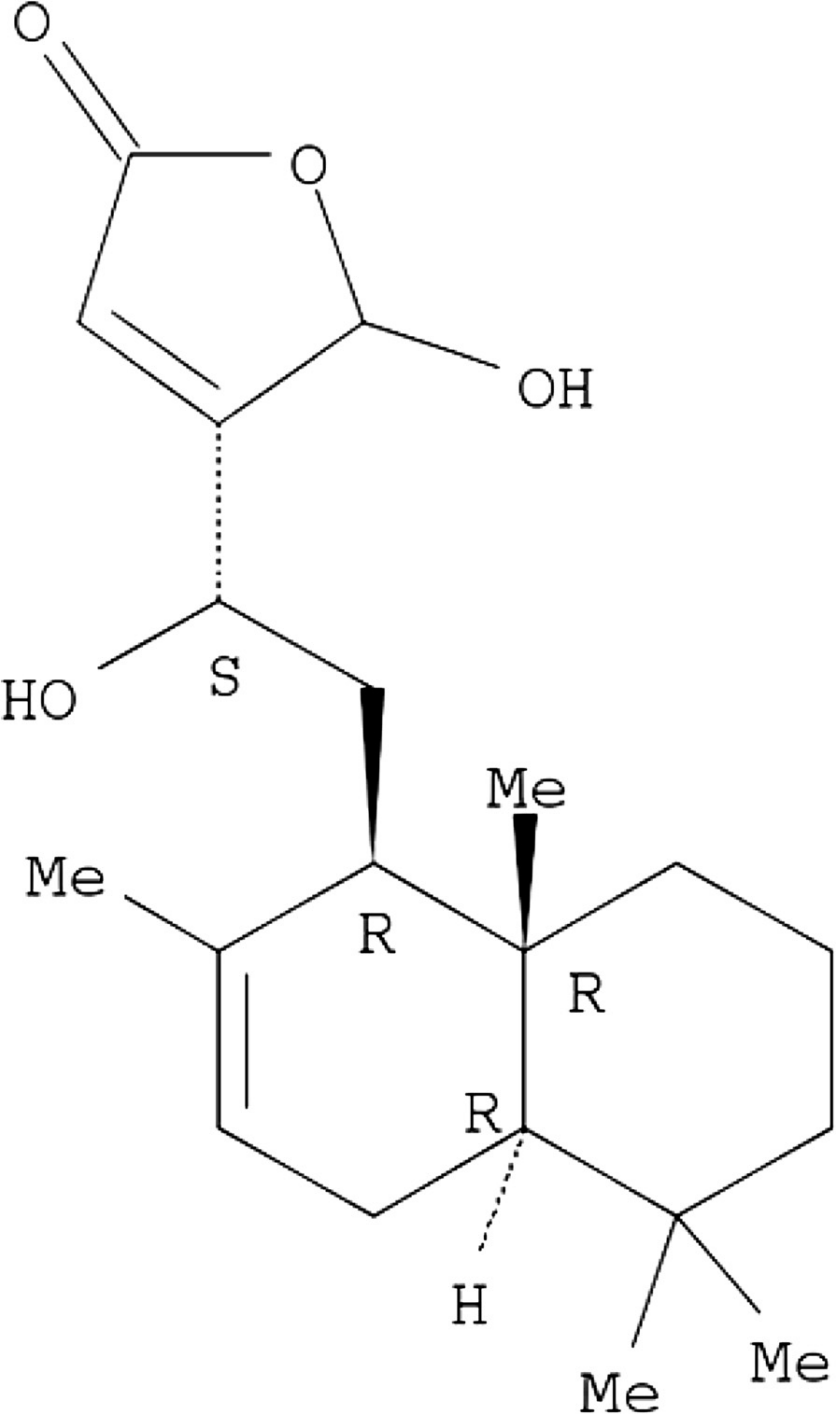
**Structure of myriadenolide.** This labdane-diterpene was isolated from *Alomia myriadenia* (Zani et al. 2000) [23].

Myriadenolide (AMY, Figure 6) was isolated from an ethanol extract of aerial parts of *Alomia myriadenia* Schultz-Bip. Ex. Baker (*Asteraceae*) (voucher code BHCB 42865) as previously described [23]. The AMY compound was dissolved in DMSO, diluted in RPMI and added to the culture to attain the desired final concentrations. Control experiments were performed using DMSO (0.005%).

### Cellular viability (MTT assay)

The cytotoxicity was determined in parallel with the antiviral activity. For that, cell viability was estimated measuring the rate of mitochondrial reduction of yellow tetrazolium salt MTT (3-(4.5-dimethylthiazol-2-yl)-2.5-diphenyltetrazolium bromide; Sigma-Aldrich, St. Louis, MO) to insoluble purple crystals [47]. After AMY incubation, MTT solution (20 μL; 5 mg MTT mL^−1^) was added to each well and incubated for 4 hours. At the end of this incubation, the supernatant was removed and 200 μL of 0.04 M HCl in isopropyl alcohol were added to dissolve the formazan crystal. The optical densities (OD) were measured with a spectrophotometer at 590 nm. Results were normalized with DMSO control (0.005%) and expressed as percentage of cell viability. Data were analyzed using Prism 5.0 (GraphPad Software Inc.).

The EC90 was determined as the concentration to reduce the viral protein expression in 90% using densitometry (measurement of the density of the viral protein bands in Western blot assay comparing with the mock treated cell [diluent control - DMSO 0.005%]) by the equation: % reduction of viral protein = 100 – (Densitometry values of treated cells ×100)/densitometry values of the control). The CC10 was defined as the cytotoxic activity that reduced the cell viability in 10%, and was measured comparing the treated cell with the mock treated cell. The equation used was: % reduction of viability = 100 – (Absorbance of treated cells x100)/Absorbance of mock treated cell. The concentrations needed to achieve 90% of reduction of viral protein expression, i.e, effective concentration (EC_90_), as well as the concentration needed to cause 10% cytotoxicity, i.e, cytotoxic concentration (CC_10_) was determinate as described [25]. Antiviral indices or selectivity index (SI) were then calculated as CC_10_/EC_90_.

### Quantification of *gag-pol* and *tax-rex* mRNA expression

RNA was extracted from MT-2 cells using Trizol (Life Technologies, Carlsbad, CA, USA), and complementary DNA was synthesized from 1 μg RNA using the High-Capacity cDNA Reverse Transcription Kit with random primers (Applied Biosystems, Foster City, USA), according to the manufacturer’s instructions. Expression levels of *gag-pol* and *tax-rex* mRNA were quantified using real-time PCR with serially diluted cDNA (250, 25, 2.5 and 0.25 ng) from HTLV-1-infected MT-2 cells to generate standard curves, and *GAPDH* was used as a reference gene. The amplification reaction for each gene (in separate tubes) was performed using cDNA synthesized from 250 ng RNA and 1X SYBR Green PCR Master Mix (Applied Biosystems, Foster City, CA, USA) in a final volume of 25 μL with 5 pM of the following primers according to Li & Green [48]: 5′- ACCAACACCATGGCCCA -3′ (sense) and 5′- GAGTCGAGGGATAAGGAAC -3′ (antisense) for *tax-rex*; 5′- GAGGGAGGAGCAAAGGTACTG -3′ (sense) and 5′- AGCCCCCAGTTCATGCAGACC -3′ (antisense) for *gag-pol*; and 5′- ACAGTCAGCCGCATCTTCTT -3′ (sense) and 5′- ACGACCAAATCCGTTGACTC -3′ (antisense) for human *GAPDH* (NM_002046.2). Real-time PCR was performed in an ABI Prism 7300 Sequence Detector System (Applied Biosystems, Foster City, CA, USA) with the following cycle conditions: 2 min at 50°C and 10 min at 95°C, followed by 45 cycles of 15 s at 95°C and 1 min at 60°C. Melting curves were performed after the end of the amplification cycles to validate the specificity of the amplified products. All standard dilutions and the control and individual samples were run in triplicate. Quantification was accepted when standard curves had slopes between –3.10 and –3.74, and the *r*^2^ was >0.99. The expression of *gag-pol* and *tax-rex* mRNA was calculated using the value of the target gene/value of GAPDH.

### Antiviral activity (viral protein reduction by immunobloting assay)

The antiviral potential of AMY against HTLV-1 was based on the inhibition of p19 and gp46 viral proteins in permanently infected MT-2 cells by immunoblotting analysis. Briefly, MT-2 cells were treated in the absence (0.05% DMSO) or presence of AMY for 24 hours (1.0, 0.01 or 0.0001 μM). Cell extracts were prepared using lysis buffer (50 mM Tris-HCl, pH 7.4, 150 mM sodium chloride, 50 mM sodium fluoride, 10 mM beta-glycerophosphate, 0.1 mM ethylenediaminetetraacetic acid, 10% glycerol, 1% Triton X-100, 1 mM phenylmethylsulfonyl fluoride, 2 mM sodium orthovanadate and 2 mg/ml each of pepstatin, leupeptin and aprotinin). Following incubation on ice for 10 min, cell extracts were clarified by centrifugation at 10,000 *g* for 20 min. Proteins were assayed using the Bradford reagent (Bio-Rad, Hercules), and 40 μg of extract from each sample was resolved by SDS-PAGE and transferred to nitrocellulose membranes (Bio-Rad Laboratories). Blots were evaluated using primary anti-HTLV-1 antibodies (monoclonal anti-p19: sc-57868; anti-gp46: sc-57865 and anti-GAPDH: sc-66163 from Santa Cruz Biotechnology), and after incubation with secondary antibody, immunoreactive bands were detected by enhanced chemiluminescence according to the manufacturer’s instructions (GE Healthcare). Densitometric analysis was performed on ImageJ software.

### Transmission electron microscopy (TEM)

MT-2 cell suspensions containing 10^6^ cells/well were cultivated with or without 1 μM AMY. Twenty-four hours post-incubation, cells were harvested by centrifugation at 400 *g* for 5 min at 4°C and washed twice with RPMI. Cells were collected and fixed in 2.5% glutaraldehyde for 18 hours. After washes in 0.1 M sodium cacodylate, fixed samples were post-fixed in 1% osmium tetroxide for 30 min. Samples were dehydrated in a graded acetone series prior to infiltration and embedding (Epon 812 resin). Ultrathin longitudinal sections (65 nm) were cut, stained with uranyl acetate and lead citrate and examined under a Tecnai G^2^ F20 electron microscope (FEI).

### Atomic force microscopy (AFM) scanning

AFM imaging was performed at room temperature on equipment monitored by a NanoScope IIId controller from Bruker AXS (Santa Barbara) operated in tapping mode and equipped with phase-imaging hardware. Commercial tapping mode tips from MikroMasch (Sofia) with 230-μm-long cantilevers, resonance frequencies of 60–90 kHz, spring constants of 2.0–5.0 N/m and a nominal tip curvature radius of 10 nm were used. Prior to microscopy, 13-mm round glass slides were washed exhaustively with neutral detergent and incubated in distilled water at 60°C overnight to remove the remaining dirt and grease. The glass slides were rinsed again with cold Milli Q water and subsequently rinsed with 100% ethanol and dried under laminar flow. After drying, 500 μl 1% poly-l-lysine was added to the slides (SIGMA–ALDRICH), and the slides were dried again under laminar flow. Cleaved mica (Ted Pella Inc) was also used for cell analysis. MT-2 cells treated with or without 1 μM and 0.001 μM AMY were centrifuged in a cytospin centrifugation apparatus (Jouan) (1000 *g*, 10 min, 4°C; 5.0 × 10^5^ cells) onto the glass slide or cleaved mica. After centrifugation, the cells were dehydrated successively with 30%, 50% and 70% ethanol. The slides were then fixed with cold methanol and dried under laminar flow. The AFM images presented here are phase and amplitude images. The sizes and heights of structures indicating HTLV-1 on the surface and viral budding were measured in 5 fields by glass slide or cleaved mica using the NanoScope software and Gwyddion software (http://gwyddion.net/) [49].

### Ethics statement

This study was conducted as an in-vitro study using cell lines. However, human PBMC was used as a control in the cell viability test and written informed consent was obtained, and this study was approved by the Ethics Committee of UFMG and Fundação HEMOMINAS.

## Abbreviations

HTLV-1: Human T-lymphotropic virus 1
ATL: Adult T-cell leukemia
HAM/TSP: HTLV-1-associated myelopathy/tropical spastic paraparesis
AMY: Labdane diterpene myriadenolide
TEM: Transmission electron microscopy
AFM: Atomic force microscopy
